# Evaluation of the *IP-10* mRNA release assay for diagnosis of TB in HIV-infected individuals

**DOI:** 10.3389/fcimb.2023.1152665

**Published:** 2023-06-02

**Authors:** Yang Tang, Yanhua Yu, Quan Wang, Zilu Wen, Ruixue Song, Yu Li, Yingquan Zhou, Ruiying Ma, Hongyan Jia, Shaoli Bai, Harimulati Abdulsalam, Boping Du, Qi Sun, Aiying Xing, Liping Pan, Jianyun Wang, Yanzheng Song

**Affiliations:** ^1^ Department of Infection and Immunity, Shanghai Public Health Clinical Center, Fudan University, Shanghai, China; ^2^ Department of Clinical Laboratory, Beijing Youan Hospital, Capital Medical University, Beijing, China; ^3^ Department of Clinical Laboratory, The Eighth Affiliated Hospital, Xinjiang Medical University, Urumqi, China; ^4^ Department of Scientific Research, Shanghai Public Health Clinical Center, Fudan University, Shanghai, China; ^5^ Beijing Chest Hospital, Capital Medical University, Beijing Key Laboratory for Drug Resistant Tuberculosis Research, Beijing Tuberculosis and Thoracic Tumor Research Institute, Beijing, China; ^6^ Department of Infectious Diseases, Gansu Provincial Infectious Disease Hospital, Lanzhou, China; ^7^ Department of Infectious Diseases, The Eighth Affiliated Hospital, Xinjiang Medical University, Urumqi, China; ^8^ Department of Geriatric Medicine, Gansu Province Hospital Rehabilitation Center, Lanzhou, China; ^9^ Department of Thoracic Surgery, Shanghai Public Health Clinical Center, Fudan University, Shanghai, China

**Keywords:** tuberculosis, M.TB infection, HIV co-infection, IP-10, mRNA, IGRA

## Abstract

HIV-infected individuals are susceptible to *Mycobacterium tuberculosis* (*M.tb*) infection and are at high risk of developing active tuberculosis (TB). Interferon-gamma release assays (IGRAs) are auxiliary tools in the diagnosis of TB. However, the performance of IGRAs in HIV-infected individuals is suboptimal, which limits clinical application. Interferon-inducible protein 10 (IP-10) is an alternative biomarker for identifying *M.tb* infection due to its high expression after stimulation with *M.tb* antigens. However, whether *IP-10* mRNA constitutes a target for the diagnosis of TB in HIV-infected individuals is unknown. Thus, we prospectively enrolled HIV-infected patients with suspected active TB from five hospitals between May 2021 and May 2022, and performed the IGRA test (QFT-GIT) alongside the *IP-10* mRNA release assay on peripheral blood. Of the 216 participants, 152 TB patients and 48 non-TB patients with a conclusive diagnosis were included in the final analysis. The number of indeterminate results of *IP-10* mRNA release assay (13/200, 6.5%) was significantly lower than that of the QFT-GIT test (42/200, 21.0%) (*P* = 0.000026). *IP-10* mRNA release assay had a sensitivity of 65.3% (95%CI 55.9% – 73.8%) and a specificity of 74.2% (95%CI 55.4% – 88.1%), respectively; while the QFT-GIT test had a sensitivity of 43.2% (95%CI 34.1% – 52.7%) and a specificity of 87.1% (95%CI 70.2% – 96.4%), respectively. The sensitivity of the *IP-10* mRNA release assay was significantly higher than that of QFT-GIT test (*P* = 0.00062), while no significant difference was detected between the specificities of these two tests (*P* = 0.198). The *IP-10* mRNA release assay showed a lower dependence on CD4^+^ T cells than that of QFT-GIT test. This was evidenced by the fact that the QFT-GIT test had a higher number of indeterminate results and a lower sensitivity when the CD4^+^ T cells counts were decreased (*P* < 0.05), while no significant difference in the number of indeterminate results and sensitivity were observed for the *IP-10* mRNA release assay among HIV-infected individuals with varied CD4^+^T cells counts (*P* > 0.05). Therefore, our study suggested that *M.tb* specific *IP-10* mRNA is a better biomarker for diagnosis of TB in HIV-infected individuals.

## Introduction

Tuberculosis (TB) is an infectious disease caused by *Mycobacterium tuberculosis* (*M.tb*) and seriously threatens human health, with an estimated 10.6 million new cases and 1.6 million deaths occurring worldwide in 2021 (WHO, 2022). However, only 63% of patients with TB were bacteriologically-confirmed. Furthermore, the diagnosis of TB is particularly difficult among HIV co-infected individuals (Scott et al., 2017; MacLean et al., 2019). Although interferon-gamma release assays (IGRAs) have been considered as a useful method for diagnosis of *M.tb* infection and an auxiliary method for diagnosis of active TB (Gao et al., 2015; Getahun et al., 2015), the sensitivity of IGRAs is reduced among immunocompromised individuals (Jung et al., 2012; Pan et al., 2015), including HIV-infected patients, which limits the clinical application of this methodology (Cattamanchi et al., 2011; Metcalfe et al., 2011; Santin et al., 2012). HIV-positive persons are particularly susceptible to *M.tb* infection, with an infection rate 2 –5 times higher than that of HIV-negative people (Mhango et al., 2021). Furthermore, the risk of developing active TB is 20 – 30 times higher for HIV and *M.tb* co-infection individuals than for those infected with *M.tb* alone. Consequently, the mortality of TB/HIV co-infected patients is also higher than that of patients with TB alone (Bell and Noursadeghi, 2018; Ignatius and Swindells, 2020). Furthermore, WHO has recommended to identify TB cases among high risk populations, such as HIV-infected individuals (WHO, 2023). Therefore, more sensitive and rapid test should be developed for the diagnosis of TB in HIV-infected individuals.

Many studies have identified an alternative biomarker for TB, namely the interferon-gamma induced protein 10 (IP-10) (Ruhwald et al., 2011; Kumar et al., 2021; Ortakoylu et al., 2022; Uzorka et al., 2022). IP-10 is expressed at a higher level than IFN-γ in the peripheral blood after exposure to *M.tb*-specific antigens (Lu et al., 2011; Ruhwald et al., 2012). Meta-analyses have shown that IP-10 detection has a sensitivity of 86% (95%CI = 80%–90%) and a specificity of 88% (95%CI = 82%–92%) in the diagnosis of active TB (Qiu et al., 2019b), and a sensitivity of 85% (95%CI = 80%–88%) and a specificity of 89% (95%CI = 84%–92%) in the diagnosis of *M.tb* infection, respectively (Qiu et al., 2019a). However, these studies have used IP-10 protein as the target, which is not detected unless the peripheral blood is stimulated overnight (18-20h) with *M.tb* antigens. This leads to the delayed reporting of diagnostic results. In contrast, the expression of *IP-10* mRNA can be up-regulated by about one hundred times within 2.5-8h of *M.tb*-specific antigen stimulation (Blauenfeldt et al., 2014). Although the sample sized was not large, recent study has showed that elevated *IP-10* at the mRNA level was also associated with pulmonary TB (Fisher et al., 2022). Thus, the development of novel diagnostic technologies, using *IP-10* mRNA as a target, may improve the speed and diagnostic sensitivity of TB testing. Such a methodology may also be more suitable for clinical use.

Our previous study has confirmed that the *IP-10* mRNA can be effectively used as a target for the diagnosis of *M.tb* infection in HIV-infected individuals, as evidenced by its higher sensitivity and lower indeterminate rate of the detection (Pan et al., 2021; Pan et al., 2022). However, whether it can be used as an auxiliary method for diagnosis of TB, should also be validated in a larger cohort. Herein, we prospectively enrolled suspected TB patients with HIV infection, and evaluated the performance of *M.tb*-specific IP-*10* mRNA release assay for TB diagnosis.

## Materials and methods

### Ethical approval

This study was performed in accordance with the guidelines of the Helsinki Declaration and was approved by the Ethics Committee of the Shanghai Public Health Clinical Center (Ethical approval number: 2020-S213-03). Written informed consents were obtained from each participant before blood collection.

### Study design and participants

This multicenter, prospective study was performed across five hospitals in China, including the Shanghai Public Health Clinical Center (Eastern), the Beijing Chest Hospital (Eastern), the Beijing Youan Hospital (Eastern), the Gansu Provincial Infectious Disease Hospital (Western) and the Eighth Affiliated Hospital, Xinjiang Medical University (Western). According to the positive rate of *IP-10* mRNA release assay (92.9%) and IGRA (61.5%) among HIV-coinfected patients in our previous study (Pan et al., 2022), we performed sample size calculation and the final minimum sample size of TB/HIV co-infected patients was 143. Therefore, we prospectively and continuously recruited the suspected HIV/TB co-infected patients from the five participating hospitals and ended when the HIV/TB co-infected patients in case group was enough, between May 2021 and May 2022. The *IP-10* mRNA release assay and QuantiFERON-TB gold In-Tube (QFT-GIT) assay were then performed in parallel using peripheral blood collected from each participant. The clinicians were blinded to the laboratory test results before the end of the clinical trial enrollment, while the laboratory technicians were blinded to the diagnosis of the patients throughout the study. Furthermore, the laboratory technicians who performed the *IP-10* mRNA release assay were unaware of QFT-GIT results, and vice versa.

### Categorization of participants

The final diagnosis was based on clinical manifestations, biochemical examinations, and the histopathological, radiological, microbiological and nucleic acid amplification information. All participants were followed up for at least 6 months to monitor whether there were changes in their diagnosis. HIV infection was defined according to the national guidelines (AIDS and Hepatitis Group of Infectious Diseases et al., 2018). Patients were categorized as having: (1) Definite TB: patients who had a positive *M.tb* culture, or positive Xpert MTB/RIF, microscopy, or histology results. (2) Probable TB: patients who had clinical and radiological evidences of TB, and presented well response to anti-TB treatment, but lacked microbiological, histopathological or nucleic acid amplification evidence of *M.tb* infection. (3) Non-TB patients: patients who has no history of TB or previous known exposure with TB, and were initially suspected of having active TB, but ended up not having active TB, due to either an alternative diagnosis was made or clinical improvement occurred without recent anti-TB therapy.

### Blood collection

A total of 10 mL of peripheral blood was collected in heparin-containing vacutainer tubes from each participant, and the *IP-10* mRNA release assay and QFT-GIT test were subsequently performed.

### The *IP-10* mRNA release assay

The commercial *IP-10* mRNA release assay was performed according to the instructions of the manufacturer, using the following kits: the kit for blood incubation and RNA extraction (CLR001A48, InnowaveDx Co.,Ltd, Suzhou, China) and the kit for reverse transcription (RT) and qPCR testing (CLR002A48, InnowaveDx Co.,Ltd, Suzhou, China), Briefly, the blood was divided into 3 tubes (1.5mL per tube): (i) one tube was coated with *M.tb*-specific peptides (ESAT6, CFP-10 and PPE68); (ii) one tube was coated with phytohemagglutinins (PHA) as a positive control; (iii) one tube had no antigen coating and was used as a negative control (Nil). The tubes were then incubated immediately for 4–6h at 37°C and the RNA was automatically extracted from the incubated whole blood in a 100 μL volume. A total of 10 μL RNA and 10 μL RT solution (composed of buffer and the reverse transcriptase) were mixed and reverse transcribed to cDNA using the following conditions: 50°C for 20 min and 85°C for 2 min. RNA extraction and RT were performed using the automatic nucleic acid extraction and detection system Innovo-100 (InnowaveDx Co.,Ltd, Suzhou, China). All the commercial reagents and kits for RNA extraction and RT were compatible with the instrument used. A total of 10 μL cDNA was then mixed with 15 μL quantitative real-time PCR (qPCR) mix (including enzyme, buffer and probe). qPCR was performed on the ABI 7500 Real-time PCR System (Thermo Fisher Scientific, Waltham, MA, USA) using the following conditions: 50°C for 2 min, 95°C for 2 min, and then 40 cycles of 95°C for 10 s and 60°C for 30 s. The cycle threshold (CT) for the target gene (*IP-10*, Gene ID: 3627) and housekeeping gene (*CHMP2A*, Gene ID: 27243) detector was automatically determined. ΔCT (the CT value for the target gene minus the CT value for the housekeeping gene) was calculated and used to determine relative gene expression as previously described (Schmittgen and Livak, 2008). The relative amount of *IP-10* mRNA in each tube (with or without antigens) was calculated separately. The test results were classified as indeterminate, negative, or positive according to the previously published criteria (Pan et al., 2022). Briefly, the test result was positive if the relative amount of IP-10 mRNA (ΔCT) in *M.tb* antigen tube minus that in Nil control tube was ≤ -1.04; the test result was negative if the ΔCT value in *M.tb* antigen tube minus that in Nil control tube was > -1.04 and the ΔCT value in mitogen tube minus that in Nil control tube was ≤ -1.2; and ‘indeterminate’ was defined as that the ΔCT value in *M.tb* antigen tube minus that in Nil control tube was > -1.04 and the ΔCT value in mitogen tube minus that in Nil control tube was > -1.2.

### The QFT-GIT test

The QFT-GIT test (QIAGEN, German) was performed in accordance with the manufacturer’s instructions. Briefly, the peripheral blood was divided into 3 tubes (1mL per tube): (i) one tube coated with *M.tb-*specific peptides (ESAT-6, CFP-10 and TB 7.7); (ii) one tube coated with the mitogen as a positive control; (iii) one tube without antigen as a negative control (Nil). The tubes were shaken about 10 times to ensure that the blood properly mixed with entire content of the tube, and then they were incubated immediately for 16–24h at 37°C. After centrifugation at 3000 × g for 15 min, the plasma IFN-γ concentration was measured immediately by ELISA according to the instructions. The test results were classified as indeterminate, negative, or positive according to the previously published criteria (Wu et al., 2018).

### Statistical analysis

Sample size calculation was performed using PASS software, version 11 (NCSS, LLC. Kaysville, Utah, USA), the parameters were set as follow: type I error (α) was 0.05, permissible error (1-β) was 0.08 and the positive rate of the tests was estimated as 61.5% as previous study (Pan et al., 2022). Data analysis was performed using SPSS for Windows, version 21 (SPSS, Inc). Categorical variables were compared by Pearson’s Chi-square test or Fisher’s exact test (when any of the sample size in the 2×2 contingency table is less than 5), while continuous variables were compared by the Student’s t-test (parametric test for analyzing the continuous variables with normal distribution) or Mann-Whitney U-test (Non-parametric test for analyzing the continuous variables without normal distribution), as appropriate. Receiver operating characteristic (ROC) curves were constructed to obtain the area under the curve (AUC) and evaluate the diagnostic value of each assay. The sensitivity and specificity of each test were also calculated to evaluate diagnostic performance. Concordance between the *IP-10* mRNA release assay and QFT-GIT assay was calculated using the Cohen’s kappa test. The criterion for statistical significance was *P* < 0.05.

## Results

### Demographic characteristics of participants

**Figure 1:**
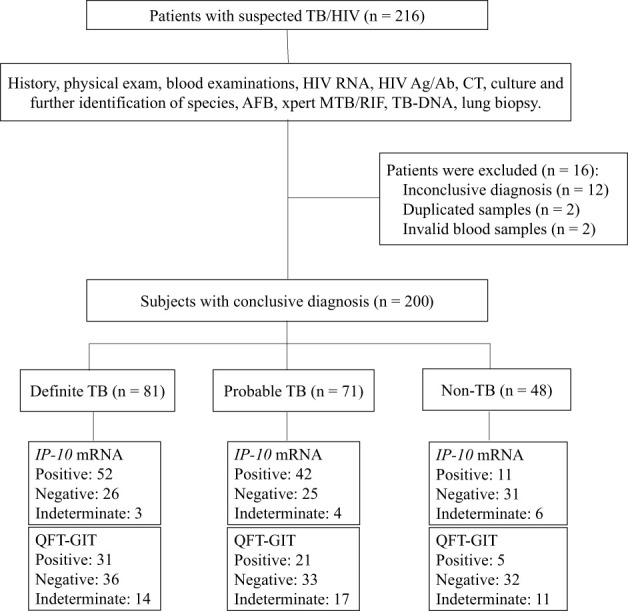
Flow chart of the study participants. Of the 216 patients with suspected TB/HIV co-infection, the following were eligible for inclusion in the final analysis: 81 were definite TB/HIV co-infected patients, 71 had probable TB/HIV co-infected patients and 48 were non-TB patients with HIV infection. TB, tuberculosis; HIV, human immunodeficiency virus; CT, computed tomography; AFB, acid-fast bacilli.

A total of 216 suspected TB patients with HIV infection were prospectively enrolled from five hospitals in China (**Figure 1**). According to their final diagnosis and a further 6-months follow-up, 81 definite TB patients with HIV infection, 71 probable TB patients with HIV infection and 48 non-TB patients with HIV infection were included in the analysis. Other 16 patients were excluded, including 12 patients who had inconclusive diagnosis, 2 patients were enrolled twice and 2 patients who had invalid peripheral blood samples. No significant differences were detected in age (*P* = 0.081) and gender (*P* = 0.056) between the TB group and non-TB group. The peripheral blood CD4^+^ T cell counts of the TB group was significantly higher than those of the non-TB group (*P* = 0.001). The detailed demographic and clinical characteristics of the participants are shown in **Table 1**.

**Table 1:** The demographic and clinical characteristics of the participants (n = 200).

	TB			Non-TB	*P*-values*
	Definite TB	Probable TB	Total TB		
Number of patients	81	71	152	48	
Age (years, range)	42 (22–72)	41 (18–79)	41 (18–29)	38 (18–63)	0.081
Gender					0.056
Male	74	67	141	48	
Female	7	4	11	0	
CD4^+^ T cell counts^§^	91 (0–587)	104 (2–1141)	102 (1–1141)	40.5 (2–336)	0.001
*M.tb* culture					
Positive	29	0	29	0	
Negative	37	62	99	34	
Not done	15	9	24	14	
Smear microscopy					
Positive	28	0	28	0	
Negative	50	68	118	44	
Not done	3	3	6	4	
Xpert MTB/RIF					
Positive	60	0	60	0	
Negative	16	64	80	35	
Not done	5	7	12	13	
Histologic results					
Positive	3	0	3	0	
Negative	2	5	7	4	
Not done	76	66	142	44	
Pulmonary TB	69	53	122	–	
Extra-pulmonary TB	12	18	30	–	
Lymph nodes	3	5	8	–	
Meninges	4	4	8	–	
Pleura	2	5	7	–	
Intestine	2	1	3		
Skeleton	0	3	3	–	
Blooddissemination	1	0	1	–	
Cause of diseases					
Pneumocystis	–	–	–	16	
Cryptococcus	–	–	–	6	
NTM diseases	–	–	–	6	
Cytomegalovirus	–	–	–	5	
Pseudomonas aeruginosa				4	
Lymphoma	–	–	–	3	
Aspergillus	–	–	–	3	
Candida albicans	–	–	–	2	
Streptococcus	–	–	–	1	
Rhodococcusequi				1	
Mucor				1	

NTM, non-tuberculous mycobacterial.

*Comparison between the total TB and non-TB group. The student’s t-test was used for the age comparison between TB and non-TB group, Fisher’s exact test was used for gender comparison between TB and non-TB group, and the Mann-Whitney U-test was used for the comparison of CD4^+^ T cell counts between TB and non-TB group.

^§^CD4^+^ T cell counts were missed among 11 TB patients and 4 non-TB patients.

### Indeterminate results of *IP-10* mRNA release assay and QFT-GIT test

Among the 200 suspected TB patients with HIV infection who were included in the final analysis, 13 patients (6.5%) had invalid results of *IP-10* mRNA release assay due to invalid ΔCT in the mitogen control tube (> -1.2), including 7 TB patients and 6 non-TB patients. Meanwhile, 42 patients (21.0%) had invalid results of QFT-GIT assay due to higher IFN-γ concentrations (> 8 IU/mL) in the Nil control tube (1 patient) or lower IFN-γ concentrations (< 0.5 IU/mL) in the positive control tube (41 patients), including 31 TB patients and 11 non-TB patients. The rate of indeterminate results of the *IP-10* mRNA release assay was significantly lower than that of QFT-GIT assay (*P* = 0.000026). Collectively, a total of 51 indeterminate results were obtained by the two assays. In addition, we found that patients with invalid results were significantly more likely to have CD4^+^ T cell counts below 200 cells/μL (43/47, 91.5%) than patients with valid results (101/138, 73.2%), suggesting that lower CD4^+^ T cell counts was a significant risk factor for indeterminate results (*P* = 0.0091).

### The concordance between the *IP-10* mRNA release assay and the QFT-GIT test

The concordance between the *IP-10* mRNA release assay and QFT-GIT assay was also analyzed in the 149 participants who had both valid results of *IP-10* mRNA release assay and QFT-GIT assay (**Table 2**). Both the *IP-10* mRNA release assay and QFT-GIT assay were positive in 44 participants and negative in 53 participants, suggesting that *IP-10* mRNA release assay and QFT-GIT assay have a weak concordance (Kappa value = 0.33, *P* < 0.001). The overall concordance between the two assays was 65.1% (95%CI *=* 57.5% – 72.8%), the positive agreement was 61.0% (95%CI= 52.2% – 69.8%) and the negative agreement was 80.6% (95%CI 66.7% – 94.6%), respectively.

**Table 2:** Analysis of the concordance between the *IP-10* mRNA release assay and the QFT-GIT assay.

		QFT-GIT		Agreement (95%CI)	Kappa value (95%CI)
		Positive	Negative		
*IP-10* mRNA release assay	Positive	44	41	65.1 (57.5 – 72.8)	0.33 (0.19 – 0.46)
	Negative	11	53		

### The diagnostic performance of the *IP-10* mRNA release assay versus QFT-GIT assay for TB

**Figure 2:**
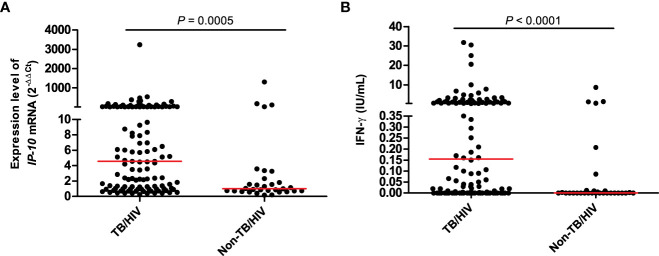
The expression level of *IP-10* mRNA in *IP-10 mRNA* release assay **(A)** and the released IFN-γ concentration in QFT-GIT assay **(B)**. TB/HIV group: n = 118; Non-TB/HIV group: n = 31; the Mann-Whitney U-test was used to perform the comparison of the expression level of *IP-10* mRNA between TB and non-TB group, as well as the comparison of IFN-γ concentration between TB and non-TB group.

The diagnostic performances of the *IP-10* mRNA release assay and QFT-GIT assay were analyzed among 118 TB patients (66 definite TB patients and 52 probable TB patients) and 31 non-TB patients, who had valid results of *IP-10* mRNA release assay and valid QFT-GIT assay (**Table 3**). The expression levels of *M.tb*-specific *IP-10* mRNA in the TB patients were significantly higher than those in the non-TB patients (**Figure 2A**). The ROC analysis showed that the AUC value for the *IP-10* mRNA release assay was 0.70 (95%CI =0.62 – 0.77), with a sensitivity of 65.3% (95%CI = 55.9% – 73.8%) and a specificity of 74.2% (95%CI = 55.4% – 88.1%). Meanwhile, the results of the QFT-GIT assay showed that the *M.tb*-specific IFN-γ concentrations were also significantly higher in the TB patients than those in the non-TB patients (**Figure 2B**). The ROC analysis showed that the AUC value for the QFT-GIT assay was 0.65 (95%CI = 0.57 – 0.72), with a sensitivity of 43.2% (95%CI = 34.1% – 52.7%) and a specificity of 87.1% (95%CI = 70.2% – 96.4), respectively. The sensitivity of the *IP-10* mRNA release assay was significantly higher than that of the QFT-GIT assay (*P* = 0.00062), while no significant difference was detected in the specificities between the *IP-10* mRNA release assay and the QFT-GIT assay (*P* = 0.198).

**Table 3:** Diagnostic performance of the *IP-10* mRNA release assay and QFT-GIT assay for TB.

	TB (N =118)	Non-TB (n = 31)	Sensitivity% (95%CI)	Specificity% (95%CI)	NPV% (95%CI)	PPV% (95%CI)	LR+ (95%CI)	LR- (95%CI)
*IP-10* mRNA release assay			65.3 (55.9–73.8)	74.2 (55.4–88.1)	35.9 (28.9–43.7)	90.6 (83.9–94.7)	2.53 (1.40–4.70)	0.47 (0.30–0.60)
Positive	77	8						
Negative	41	23						
QFT-GIT			43.2 (34.1–52.7)	87.1 (70.2–96.4)	28.7 (24.7–33.2)	92.7 (83.3–97.0)	3.35 (1.30–8.60)	0.65 (0.50–0.80)
Positive	51	4						
Negative	67	27						
*IP-10* or QFT-GIT *			73.7 (64.8–81.4)	71.0 (52.0–85.8)	41.5 (32.7–50.8)	90.6 (84.7–94.4)	2.54 (1.40–4.40)	0.37 (0.30-0.50)
Positive	87	9						
Negative	31	22						

NPV, negative predictive value; PPV, positive predictive value; LR+, likelihood ratio for positive test; LR−, likelihood ratio for negative value.

*A result was considered positive when either test was positive, and a result was deemed negative when both tests were negative.

The diagnostic performance of the combination of *IP-10* mRNA release assay and QFT-GIT assay was also evaluated, and the positive result was assumed when either test was positive and a negative result was deemed when both tests were negative (**Table 3**). Based on this standard, the diagnostic sensitivity and specificity were 73.7% (95%CI = 64.8%– 81.4%) and 71.0% (95%CI = 52.0%– 85.8%), respectively. In comparison with the QFT-GIT assay alone, the combination of tests significantly increased the sensitivity of TB detection by 30.5%, although this was accompanied by a 16.1% decrease in the specificity. However, the highly improved sensitivity may be of great benefit for identifying of TB in HIV-infected individuals.

Subgroup analyses were also performed in definite TB group and probable TB group (**Supplementary Table 1**). The sensitivities of the *IP-10* mRNA release assay and the QFT-GIT assay in the definite TB group were 68.2% (95%CI = 55.6%– 79.1%)and 47.0% (95%CI = 34.6%– 59.7%), respectively. The sensitivities of the *IP-10* mRNA release assay and the QFT-GIT assay in the probable TB group were 61.5% (95%CI = 47.0%– 74.7%)and 38.5% (95%CI = 25.3%– 53.0%), respectively. Significant differences were detected in the sensitivity between the *IP-10* mRNA release assay and the QFT-GIT assay, in both definite TB group (*P* = 0.0137) and probable TB group (*P* = 0.0186). However, there is no significant differences in the sensitivity between definite TB group and probable TB group, either in the *IP-10* mRNA release assay (*P* = 0.452) or in the QFT-GIT assay (*P* = 0.354).

### The impact of CD4^+^ T cell exhaustion on the *IP-10* mRNA release assay and the QFT-GIT assay

**Figure 3:**
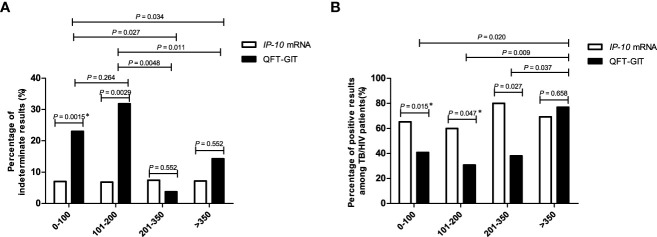
The percentages of indeterminate results and positive results of the *IP-10* mRNA release assay and the QFT-GIT assay in HIV-infected patients with different CD4^+^ T-cell counts. **(A)** The percentages of indeterminate results obtained via each of the tests in the cohort of HIV-infected individuals enrolled in this study. The number of patients with different CD4^+^ T cells were described as follow: ≤ 100/μL,n = 100; 101-200/μL, n = 44; 201-350/μL, n = 27; > 350/μL, n = 14. The CD4^+^ T cell counts were missed in 15 patients. *Pearson’s Chi-square test was used for comparison of the percentage of indeterminate results between *IP-10* mRNA release assay and QFT-GIT in the patients with CD4^+^ T cells counts less than 100/μL. Other comparisons were performed using Fisher’s exact test between patients with different CD4^+^ T cells counts, or between *IP-10* mRNA release assay and QFT-GIT. **(B)** The percentages of positive results obtained by each of the tests among TB/HIV co-infected individuals. The number of TB/HIV co-infected patients with different CD4^+^ T cells were described as follow: ≤ 100/μL, n = 49; 101-200/μL, n = 25; 201-350/μL, n = 20; > 350/μL, n = 13. The CD4^+^ T cell counts were missed in 11 TB/HIV co-infected patients. *Pearson’s Chi-square test was used for comparison of the percentage of positive results between *IP-10* mRNA release assay and QFT-GIT in the patients with CD4^+^ T cells counts less than 100/μL, as well as in the patients with CD4^+^ T cells counts range from 101/μL to 200/μL. Other comparisons were performed using Fisher’s exact test between patients with different CD4^+^ T cells counts, or between *IP-10* mRNA release assay and QFT-GIT.

Both the *IP-10* mRNA release assay and QFT-GIT assay function on the basis of the lymphocyte response to *M.tb*-specific antigens. Since CD4^+^ T cells exhaustion commonly occurred in HIV-infected individuals, we analyzed whether CD4^+^ T cell exhaustion could affect the outcomes of the two tests. The indeterminate results rate and positive result rate of the two tests were calculated for the HIV-infected individuals with different amount of CD4^+^ T cell counts. As shown in **Figure 3A**, there was no significant difference in the indeterminate result rate between the *IP-10* mRNA release assay and the QFT-GIT assay among HIV-positive patients with CD4^+^ T cell counts > 200 cells/μL. However, the indeterminate result rates of the *IP-10* mRNA release assay were significantly lower than those of the QFT-GIT assay among the patients with CD4^+^ T cell counts < 200 cells/μL (≤ 100 cells/μL: *P* = 0.0015; 101-200 cells/μL: *P* = 0.0029). No significant difference in the indeterminate result rate of the *IP-10* mRNA release assay was detected between patients with different amount of CD4^+^T cells (*P* > 0.05), suggesting that there was no significant impact of CD4^+^T cells exhaustion on the results of the *IP-10* mRNA release assay. However, the indeterminate result rate of the QFT-GIT assay was significantly higher in patients with CD4^+^ T cell counts < 200 cells/μL than those in patients with CD4^+^ T cell counts > 200 cells/μL (*P*= 0.012), suggesting that CD4^+^ T cells exhaustion had a significant impact on the performance of the QFT-GIT assay.

As shown in **Figure 3B**, the positive result rate of the *IP-10* mRNA release assay was significantly higher than that of the QFT-GIT assay among TB/HIV co-infected patients with CD4^+^ T cell counts < 350 cells/μL (≤ 100 cells/μL: *P* = 0.015; 101-200 cells/μL: *P* = 0.047; 201-350cells/μL: *P* = 0.027). Furthermore, no significant difference in the positive result rate of the *IP-10* mRNA release assay was detected between TB/HIV co-infected patients with different amounts of CD4^+^ T cells (*P* > 0.05), also indicating that there was no significant impact of CD4^+^ T cells exhaustion on the performance of the *IP-10* mRNA release assay. Nevertheless, the positive result rate of the QFT-GIT assay was significantly higher in the TB/HIV co-infected patients with CD4^+^ T cell counts > 350 cells/μL than those in the patients with CD4^+^ T cell counts < 350 cells/μL (≤ 100 cells/μL *vs.*> 350 cells/μL: *P* = 0.020; 101-200 cells/μL *vs.*> 350 cells/μL: *P* = 0.009; 201-350 cells/μL *vs.*> 350 cells/μL: *P* = 0.037), indicating that CD4^+^ T cells exhaustion had a significant impact on the outcome of the QFT-GIT assay.

## Discussion

The identification of TB cases and the implementation of anti-TB treatment in high risk population, including HIV-infected individuals, have been recommended by the WHO (WHO, 2023). However, the utility of traditional immunological tests (e. g. tuberculin test and IGRAs) has been limited for auxiliary TB diagnosis in immunocompromised individuals (Cattamanchi et al., 2011). In this study, we used *IP-10* mRNA as a target due to its higher transient expression levels after stimulation with *M.tb*-specific antigens. We found that the *IP-10* mRNA release assay had a positive rate (65.3%) for the detection of TB in HIV co-infected individuals that was more than 20% higher than that of the conventional QFT-GIT assay. This result was consistent with previous studies that used the IP-10 protein as a target. Data from countries with a moderate or low TB burden suggested that when used as a target, the IP-10 protein yielded a higher positive rate (66.7%) of diagnosing *M. tb* infection than the IGRAs (52.4%) among HIV-infected persons. Furthermore, the outcome of the IP-10 protein detection test is not influenced by the reduction in CD4^+^ T cell counts due to HIV-mediated exhaustion (Vanini et al., 2012). It was also reported that the IP-10 protein test had a higher positive rate (45.0% *vs*. 38.0%) and a lower indeterminate rate (5.0% *vs*. 9.0%) than the IGRAs test in HIV-infected individuals from a country with a higher TB burden (Kabeer et al., 2011). Another study has shown that IP-10 protein instead of IFN-γ yielded higher positive rate either after stimulation with commercial *M.tb* antigens in IGRA test (85.7% *vs*. 60.7%) or stimulation with in-house *M.tb* antigens (75.0% *vs*. 42.9%) (Goletti et al., 2010). Our results and these previous reports collectively indicated that either the IP-10 protein or its mRNA could improve the identification of TB among HIV-infected persons. We envisage that this strategy will help early screen of active TB in immuno-compromised individuals.

The higher positive rate of the *IP-10* mRNA release assay in TB/HIV co-infected individuals may be due to the higher expression level of *IP-10* mRNA after *M.tb* specific antigen stimulation. As a downstream amplification molecule of IFN-γ-mediated signaling, IP-10 is more highly expressed than IFN-γ (Tsuboi et al., 2011). On the other hand, IP-10 is not only released in response to IFN-γ stimulation, but is also induced by other cytokines, including IFN-α, IL-17 and IL-23 (Khader et al., 2007; Simmons et al., 2013). Furthermore, IP-10 is mainly released by monocytes, meaning that the impact of CD4^+^ T cell exhaustion on the outcome of the *IP-10* mRNA release assay will be lower (Wu et al., 2017).

The specificity of the *IP-10* mRNA release assay was lower than that of QFT-GIT assay in non-TB patients, although the difference did not reach statistical significance. Since the study design involved the prospective enrollment of highly suspected TB patients with HIV infection, there were only 48 non-TB patients enrolled in the study. Of these non-TB patients, 31 had both valid results of the *IP-10* mRNA release assay and the QFT-GIT assay. The small size of the non-TB patients may lead to the enlarged difference between the specificities of these two tests. Our previous study found that there was no significant difference in specificity between the *IP-10* mRNA release assay and the IGRA assay among patients without HIV infection (Pan et al., 2022). Furthermore, Vanini et al. also reported that although IP-10 protein as a target yielded a slight higher positive result rate than that IFN-γ as a target in the non-TB patients with HIV infection, there were no significant differences between the use of IP-10 and IFN-γ as a target either in individuals with a higher risk of *M.tb* infection (IP-10 40.0%*vs.* IFN-γ 37.5%) or in individuals with a lower risk of *M.tb* infection (IP-10 12.9%*vs.* IFN-γ 4.8%) (Vanini et al., 2012). However, given that there is no gold standard test for latent *M.tb* infection, we cannot determine whether those patients with negative IGRA results but positive IP-10 results are *M.tb* infection or not. Therefore, we should also pay attention to the abovementioned patients and performed further exploration in a larger sample set. In addition, the positive results of these two tests in non-TB patients may be caused by the higher rate of latent TB infection (LTBI) in China. The previously estimated prevalence of LTBI in China was about 20.3% among persons older than 15 years, based on the results of the IGRA tests (Gao et al., 2015; Institute of Pathogen Biology, CAMS, et al., 2022). This value is consistent with the positive result rate in the non-TB patients in our study. Since, like IGRAs, the *IP-10* mRNA release assay is also a method for the diagnosis of *M.tb* infection and auxiliary method for diagnosis of TB, some positive results within the non-TB patient group were also expected.

The *IP-10* mRNA release assay and the QFT-GIT assay both detect the host immune response to antigen stimulation. Due to the dysfunctional immune system of HIV-infected persons, some of the patients presented no response to the PHA positive control, which was the main reason for the indeterminate results. Our data also showed that a significantly higher number of patients with CD4^+^ T cell counts below 200 cells/μL had invalid test results. Nevertheless, the indeterminate result rate of the *IP-10* mRNA release assay was significantly lower than that of the QFT-GIT assay, which may be caused by the different antigens used in the two tests. There may also be another reason for the stronger response to *M.tb*-specific antigens exhibited by the *IP-10* mRNA release assay; the higher expression level of the *IP-10* mRNA may lead to more positive results (and fewer indeterminate ones) even in the absence of a response to the PHA positive control.

In the present study, the concordance between the *IP-10* mRNA release assay and QFT-GIT assay was low. This may be due to the different targets (IP-10 *vs.* IFN-γ) and the different expression level (mRNA *vs*. protein) between the two tests. Furthermore, the *M.tb-*specific antigens were different between the two tests. The antigens in the QFT-GIT assay were ESAT-6, CFP-10 and TB7.7, while the antigens in the *IP-10* mRNA release assay were ESAT6, CFP-10 and PPE68. It is worth noting that the negative concordance rate between the two tests in non-TB patients was better than the positive concordance rate between the two tests in TB patients. This lower positive concordance rate mainly due to the lower sensitivity of the QFT-GIT test and represents a further significant difference between the two tests in HIV-infected individuals.

The results of the QFT-GIT test were influenced by CD4^+^ T cells exhaustion, while those of the *IP-10* mRNA release assay were not. When the CD4^+^ T cell counts were at a normal level (>350 cells/μL), there was no significant difference between the indeterminate rate or sensitivity of the *IP-10* mRNA release assay and the QFT-GIT assay. These results were consistent with our previous study involving non-HIV individuals (Pan et al., 2022). However, the indeterminate result rate of the QFT-GIT assay was higher and the sensitivity was lower when CD4^+^ T cell counts decreased. This is likely because HIV infection impairs CD4^+^ T cell proliferation and function (Day et al., 2017; Amelio et al., 2019) and leads to dysfunctional Th1 immune response, which reduces IFN-γ release. It is currently believed that the reduction in IFN-γ release due to the depletion of *M.tb*-specific Th1 cells during HIV infection is a typical mechanism by which the virus damages host defense against *M.tb* (Kalsdorf et al., 2009). The lesser impact of CD4^+^ T cells exhaustion on the performance of the *IP-10* mRNA release assay indicates that the release of *IP-10* mRNA may not be completely dependent on CD4^+^ T cells. Flow cytometry was used to shown that the expression of high levels of *IP-10* mRNA in HIV-infected individuals was mainly a feature of monocytes and myeloid dendritic cells (mDCs) (Rempel et al., 2010; Simmons et al., 2013). Although *IP-10* mRNA in T lymphocytes was also up-regulated after HIV infection, it gradually decreased following antiviral treatment initiation (Foley et al., 2005). Therefore, unlike the release kinetics of IFN-γ, *IP-10* mRNA release may not depend on T lymphocytes and thus is less affected by the depletion of CD4^+^ T cells induced by HIV infection. Furthermore, these results also suggested that the combined use of the *IP-10* mRNA release assay and conventional IGRAs assay could identify more individuals with TB.

Apart from the better performance of *IP-10* mRNA release assay in HIV co-infected populations, previously we also found that the performance of *IP-10* mRNA release assay was similar with IGRA in patients without HIV co-infection, indicating the wider application of *IP-10* mRNA release assay in clinical practice (Pan et al., 2022). Furthermore, there are some other advantages. Firstly, The higher expression level of *IP-10* mRNA after *M.tb* antigen stimulation leads to higher sensitivity in diagnosis, and it is better to avoid the adverse effects of non-specific IP-10 release by other infections or background IP-10 release. Furthermore, the whole procedures of *IP-10* mRNA release assay are automatic and this will reduce the bias by manual operation. In addition, the detection linear range of the PCR technique is wider than that of ELISA test, and it does not rely on a standard curve to determine concentrations. Lastly, after covid-19 pandemic, the PCR technique is already an integrated tool in most TB laboratories and the cost is reduced due to the widely use in clinical practice, so the PCR platform used for diagnosis is reasonable. In the present study, we prospectively evaluate the diagnostic performance of the *IP-10* mRNA release assay in the diagnosis of TB in a cohort of HIV-infected individuals. However, our research has limitations. Firstly, it would have been beneficial to validate the inconsistencies between the *IP-10* mRNA release assay and QFT-GIT test results using a third method. However, the fact that the positive result rate of the *IP-10* mRNA release assay was significantly higher than that of the QFT-GIT assay in the group of patients with definite TB (who had microbiological evidence for *M.tb* infection) indirectly indicates the better performance of the *IP-10* mRNA release assay. Secondly, our study only prospectively enrolled patients from multiple centers within one year. This meant that the sample size was not large enough to evaluate of the performance of IP-10 for diagnosing TB in HIV-infected individuals, although it is larger than previous studies. Further validation in a larger sample set would yield more convincing evidence. Thirdly, we did not analyze the impact of the antiretroviral therapy on the performance of the two tests due to the limited sample size. In future, an in-depth analysis of diagnostic performance of *IP-10* mRNA release assay for TB in patients receiving different forms or duration of antiretroviral therapy will facilitate for reasonable utility of this novel test. Finally, this study was performed in adults. Whether the *IP-10* mRNA release assay performs better in immunocompromised children with suspected TB should be validated in future studies.

In conclusion, our study has evaluated side by side the performance of the two tests which are based on whole blood antigen stimulation, and confirmed that the *IP-10* mRNA release assay was superior at detecting TB in HIV-infected patients than the conventional QFT-GIT test. The higher sensitivity and lower indeterminate result rate of the *IP-10* mRNA release assay will likely aid the early detection of TB in this higher risk population of immunocompromised individuals.

## Data availability statement

The raw data supporting the conclusions of this article will be made available by the authors, without undue reservation.

## Ethics statement

The studies involving human participants were reviewed and approved by the Ethics Committee of the Shanghai Public Health Clinical Center. The patients/participants provided their written informed consent to participate in this study.

## Author contributions

YS, LP, JW, YY and QW designed the experiments. YT, RS, YL, YZ, RM, BP and QS conducted the experiments. YY, ZW, HA, HJ, SB and AX enrolled the subjects. LP and YT analyzed the data. LP wrote the paper. All authors contributed to the article and approved the submitted version.

## References

[B1] AIDS and Hepatitis Group of Infectious Diseases Branch of Chinese Medical Association Chinese Center for Disease Control and Prevention (2018). Guidelines for AIDS diagnosis and treatment in China. Chin. J. Infect. Dis. 36 (12), 705–724. doi: 10.3760/cma.j.issn.1000-6680.2018.12.001

[B2] Amelio P. Portevin D. Hella J. Reither K. Kamwela L. Lweno O.. (2019). HIV Infection functionally impairs mycobacterium tuberculosis-specific CD4 and CD8 T-cell responses. J. Virol. 93 (5), e01728-18. doi: 10.1128/JVI.01728-18 30541853 PMC6384080

[B3] Bell L. C. K. Noursadeghi M. (2018). Pathogenesis of HIV-1 and mycobacterium tuberculosis co-infection. Nat. Rev. Microbiol. 16 (2), 80–90. doi: 10.1038/nrmicro.2017.128 29109555

[B4] Blauenfeldt T. Heyckendorf J. Graff Jensen S. Lange C. Drabe C. Hermansen T. S.. (2014). Development of a one-step probe based molecular assay for rapid immunodiagnosis of infection with m. tuberculosis using dried blood spots. PloS One 9 (9), e105628. doi: 10.1371/journal.pone.0105628 25184553 PMC4153573

[B5] Cattamanchi A. Smith R. Steingart K. R. Metcalfe J. Z. Date A. Coleman C.. (2011). Interferon-gamma release assays for the diagnosis of latent tuberculosis infection in HIV-infected individuals: a systematic review and meta-analysis. J. Acquir. Immune Defic. Syndr. 56 (3), 230–238. doi: 10.1097/QAI.0b013e31820b07ab 21239993 PMC3383328

[B6] Day C. L. Abrahams D. A. Harris L. D. van Rooyen M. Stone L. de Kock M.. (2017). HIV-1 infection is associated with depletion and functional impairment of mycobacterium tuberculosis-specific CD4 T cells in individuals with latent tuberculosis infection. J. Immunol. 199 (6), 2069–2080. doi: 10.4049/jimmunol.1700558 28760884 PMC5624214

[B7] Fisher K. L. Moodley D. Rajkumar-Bhugeloo K. Baiyegunhi O. O. Karim F. Ndlovu H.. (2022). Elevated IP-10 at the protein and gene level associates with pulmonary TB. Front. Cell Infect. Microbiol. 12, 908144. doi: 10.3389/fcimb.2022.908144 35694534 PMC9184682

[B8] Foley J. F. Yu C. R. Solow R. Yacobucci M. Peden K. W. Farber J. M. (2005). Roles for CXC chemokine ligands 10 and 11 in recruiting CD4+ T cells to HIV-1-infected monocyte-derived macrophages, dendritic cells, and lymph nodes. J. Immunol. 174 (8), 4892–4900. doi: 10.4049/jimmunol.174.8.4892 15814716

[B9] Gao L. Lu W. Bai L. Wang X. Xu J. Catanzaro A.. (2015). Latent tuberculosis infection in rural China: baseline results of a population-based, multicentre, prospective cohort study. Lancet Infect. Dis. 15 (3), 310–319. doi: 10.1016/S1473-3099(14)71085-0 25681063

[B10] Getahun H. Matteelli A. Chaisson R. E. Raviglione M. (2015). Latent mycobacterium tuberculosis infection. N Engl. J. Med. 372 (22), 2127–2135. doi: 10.1056/NEJMra1405427 26017823

[B11] Goletti D. Raja A. Syed Ahamed Kabeer B. Rodrigues C. Sodha A. Carrara S.. (2010). Is IP-10 an accurate marker for detecting m. tuberculosis-specific response in HIV-infected persons? PloS One 5 (9), e12577. doi: 10.1371/journal.pone.0012577 20830287 PMC2935361

[B12] Ignatius E. H. Swindells S. (2020). Are we there yet? short-course regimens in TB and HIV: from prevention to treatment of latent to XDR TB. Curr. HIV/AIDS Rep. 17 (6), 589–600. doi: 10.1007/s11904-020-00529-8 32918195 PMC9178518

[B13] Institute of Pathogen Biology China Academy of Medical Sciences and Peking Union Medical College China Center for Disease Control and Prevention Union Medical Institute of Geographic Sciences and Natural Resources Research Chinese Academy of Sciences (2022). Expert consensus on the estimation of the national burden on latent tuberculosis infection. Chin. J. Antituberculosis 44 (1), 4–8. doi: 10.19982/j.issn.1000-6621.20210662

[B14] Jung J. Y. Lim J. E. Lee H. J. Kim Y. M. Cho S. N. Kim S. K.. (2012). Questionable role of interferon-gamma assays for smear-negative pulmonary TB in immunocompromised patients. J. Infect. 64 (2), 188–196. doi: 10.1016/j.jinf.2011.09.008 22120597

[B15] Kabeer B. S. Sikhamani R. Raja A. (2011). Comparison of interferon gamma-inducible protein-10 and interferon gamma-based QuantiFERON TB gold assays with tuberculin skin test in HIV-infected subjects. Diagn. Microbiol. Infect. Dis. 71 (3), 236–243. doi: 10.1016/j.diagmicrobio.2011.07.012 21996360 PMC3193601

[B16] Kalsdorf B. Scriba T. J. Wood K. Day C. L. Dheda K. Dawson R.. (2009). HIV-1 infection impairs the bronchoalveolar T-cell response to mycobacteria. Am. J. Respir. Crit. Care Med. 180 (12), 1262–1270. doi: 10.1164/rccm.200907-1011OC 19797156 PMC2796736

[B17] Khader S. A. Bell G. K. Pearl J. E. Fountain J. J. Rangel-Moreno J. Cilley G. E.. (2007). IL-23 and IL-17 in the establishment of protective pulmonary CD4+ T cell responses after vaccination and during mycobacterium tuberculosis challenge. Nat. Immunol. 8 (4), 369–377. doi: 10.1038/ni1449 17351619

[B18] Kumar N. P. Hissar S. Thiruvengadam K. Banurekha V. V. Suresh N. Shankar J.. (2021). Discovery and validation of a three-cytokine plasma signature as a biomarker for diagnosis of pediatric tuberculosis. Front. Immunol. 12, 653898. doi: 10.3389/fimmu.2021.653898 33936077 PMC8085486

[B19] Lu C. Wu J. Wang H. Wang S. Diao N. Wang F.. (2011). Novel biomarkers distinguishing active tuberculosis from latent infection identified by gene expression profile of peripheral blood mononuclear cells. PloS One 6 (8), e24290. doi: 10.1371/journal.pone.0024290 21904626 PMC3164189

[B20] MacLean E. Saravu K. Pai M. (2019). Diagnosing active tuberculosis in people living with HIV: an ongoing challenge. Curr. Opin. HIV AIDS 14 (1), 46–54. doi: 10.1097/COH.0000000000000512 30346311

[B21] Metcalfe J. Z. Everett C. K. Steingart K. R. Cattamanchi A. Huang L. Hopewell P. C.. (2011). Interferon-gamma release assays for active pulmonary tuberculosis diagnosis in adults in low- and middle-income countries: systematic review and meta-analysis. J. Infect. Dis. 204 Suppl 4, S1120–S1129. doi: 10.1093/infdis/jir410 21996694 PMC3192542

[B22] Mhango D. V. Mzinza D. T. Jambo K. C. Mwandumba H. C. (2021). New management approaches to tuberculosis in people living with HIV. Curr. Opin. Infect. Dis. 34 (1), 25–33. doi: 10.1097/QCO.0000000000000704 33315751 PMC9608335

[B23] Ortakoylu M. G. Bahadir A. Iliaz S. Soy Bugdayci D. Uysal M. A. Paker N.. (2022). Interferon-inducible protein-10 as a marker to detect latent tuberculosis infection in patients with inflammatory rheumatic diseases. J. Pers. Med. 12 (7), 1027. doi: 10.3390/jpm12071027 35887523 PMC9318865

[B24] Pan L. Gao M. Jia H. Huang M. Wei R. Sun Q.. (2021). Diagnostic performance of a novel mycobacterium tuberculosis specific T-cell based assay for tuberculosis. Chin. J. Tuberculosis Respir. Dis. 44 (5), 1–7. doi: 10.3760/cma.j.cn112147-20200821-00916 34865364

[B25] Pan L. Huang M. Jia H. Deng G. Chen Y. Wei R.. (2022). Diagnostic performance of a novel CXCL10 mRNA release assay for mycobacterium tuberculosis infection. Front. Microbiol. 13, 825413. doi: 10.3389/fmicb.2022.825413 35432271 PMC9005954

[B26] Pan L. Jia H. Liu F. Sun H. Gao M. Du F.. (2015). Risk factors for false-negative T-SPOT.TB assay results in patients with pulmonary and extra-pulmonary TB. J. Infect. 70 (4), 367–380. doi: 10.1016/j.jinf.2014.12.018 25597825

[B27] Qiu X. Tang Y. Yue Y. Zeng Y. Li W. Qu Y.. (2019a). Accuracy of interferon-gamma-induced protein 10 for diagnosing latent tuberculosis infection: a systematic review and meta-analysis. Clin. Microbiol. Infect. 25 (6), 667–672. doi: 10.1016/j.cmi.2018.12.006 30553864

[B28] Qiu X. Xiong T. Su X. Qu Y. Ge L. Yue Y.. (2019b). Accumulate evidence for IP-10 in diagnosing pulmonary tuberculosis. BMC Infect. Dis. 19 (1), 924. doi: 10.1186/s12879-019-4466-5 31666025 PMC6822474

[B29] Rempel H. Sun B. Calosing C. Pillai S. K. Pulliam L. (2010). Interferon-alpha drives monocyte gene expression in chronic unsuppressed HIV-1 infection. AIDS 24 (10), 1415–1423. doi: 10.1097/QAD.0b013e32833ac623 20495440 PMC2991092

[B30] Ruhwald M. Aabye M. G. Ravn P. (2012). IP-10 release assays in the diagnosis of tuberculosis infection: current status and future directions. Expert Rev. Mol. Diagn. 12 (2), 175–187. doi: 10.1586/erm.11.97 22369377

[B31] Ruhwald M. Dominguez J. Latorre I. Losi M. Richeldi L. Pasticci M. B.. (2011). A multicentre evaluation of the accuracy and performance of IP-10 for the diagnosis of infection with m. tuberculosis. Tuberculosis (Edinb) 91 (3), 260–267. doi: 10.1016/j.tube.2011.01.001 21459676

[B32] Santin M. Munoz L. Rigau D. (2012). Interferon-gamma release assays for the diagnosis of tuberculosis and tuberculosis infection in HIV-infected adults: a systematic review and meta-analysis. PloS One 7 (3), e32482. doi: 10.1371/journal.pone.0032482 22403663 PMC3293815

[B33] Schmittgen T. D. Livak K. J. (2008). Analyzing real-time PCR data by the comparative C(T) method. Nat. Protoc. 3 (6), 1101–1108. doi: 10.1038/nprot.2008.73 18546601

[B34] Scott L. da Silva P. Boehme C. C. Stevens W. Gilpin C. M. (2017). Diagnosis of opportunistic infections: HIV co-infections - tuberculosis. Curr. Opin. HIV AIDS 12 (2), 129–138. doi: 10.1097/COH.0000000000000345 28059955 PMC6024079

[B35] Simmons R. P. Scully E. P. Groden E. E. Arnold K. B. Chang J. J. Lane K.. (2013). HIV-1 infection induces strong production of IP-10 through TLR7/9-dependent pathways. AIDS 27 (16), 2505–2517. doi: 10.1097/01.aids.0000432455.06476.bc 24096630 PMC4288813

[B36] Tsuboi H. Wakamatsu E. Iizuka M. Nakamura Y. Sugihara M. Suzuki T.. (2011). Importance of serine727 phosphorylated STAT1 in IFNgamma-induced signaling and apoptosis of human salivary gland cells. Int. J. Rheum Dis. 14 (1), 86–91. doi: 10.1111/j.1756-185X.2010.01575.x 21303487

[B37] Uzorka J. W. Bakker J. A. van Meijgaarden K. E. Leyten E. M. S. Delfos N. M. Hetem D. J.. (2022). Biomarkers to identify mycobacterium tuberculosis infection among borderline QuantiFERON results. Eur. Respir. J. 60 (2), 2102665. doi: 10.1183/13993003.02665-2021 35058249 PMC9363845

[B38] Vanini V. Petruccioli E. Gioia C. Cuzzi G. Orchi N. Rianda A.. (2012). IP-10 is an additional marker for tuberculosis (TB) detection in HIV-infected persons in a low-TB endemic country. J. Infect. 65 (1), 49–59. doi: 10.1016/j.jinf.2012.03.017 22465752

[B39] World health organization (2022). Global tuberculosis report 2021 (Geneva: World Health Organization).

[B40] World health organization (2023). WHO standard: universal access to rapid tuberculosis diagnostics (Geneva: World Health Organization).

[B41] Wu U. I. Chuang Y. C. Sheng W. H. Sun H. Y. Jhong Y. T. Wang J. Y.. (2018). Use of QuantiFERON-TB gold in-tube assay in screening for neutralizing anti-interferon-gamma autoantibodies in patients with disseminated nontuberculous mycobacterial infection. Clin. Microbiol. Infect. 24 (2), 159–165. doi: 10.1016/j.cmi.2017.06.029 28694201

[B42] Wu X. Zhang L. L. Yin L. B. Fu Y. J. Jiang Y. J. Ding H. B.. (2017). Deregulated MicroRNA-21 expression in monocytes from HIV-infected patients contributes to elevated IP-10 secretion in HIV infection. Front. Immunol. 8, 1122. doi: 10.3389/fimmu.2017.01122 28955339 PMC5601991

