# Supplementary material for: Evaluation of the IP-10 mRNA release assay for diagnosis of TB in HIV-infected individuals
Source: Front Cell Infect Microbiol. 2023 Jun 2;13:1152665. doi: 10.3389/fcimb.2023.1152665 (PMC10272546; doi:10.3389/fcimb.2023.1152665)
Supplement: Supplementary file 1 [file Table_1.docx]

**Supplementary Table 1. Diagnostic performance of the *IP-10* mRNA release assay and QFT-GIT assay in subgroups**

|  | ***IP-10* mRNA release assay** | | |  | **QFT-GIT assay** | | | ***P*-value*** |
| --- | --- | --- | --- | --- | --- | --- | --- | --- |
|  | **Positive** | **Negative** | **Sensitivity% (95% CI)** |  | **Positive** | **Negative** | **Sensitivity% (95% CI)** |  |
| Definite TB (n = 66) | 45 | 21 | 68.2 (55.6 – 79.1) |  | 31 | 35 | 47.0 (34.6 – 59.7) | 0.0137 |
| Probable TB (n = 52) | 32 | 20 | 61.5 (47.0 – 74.7) |  | 20 | 32 | 38.5 (25.3% – 53.0) | 0.0186 |

* Comparison between *IP-10* mRNA release assay and QFT-GIT assay in both definite TB and probable TB groups.
